# Exploring the association between circRNA expression and pediatric obesity based on a case–control study and related bioinformatics analysis

**DOI:** 10.1186/s12887-023-04261-1

**Published:** 2023-11-13

**Authors:** Guobo Li, Xingyan Xu, Le Yang, Yingying Cai, Yi Sun, Jianhui Guo, Yawen Lin, Yuduan Hu, Mingjun Chen, Huangyuan Li, Siying Wu

**Affiliations:** 1 Department of Child Healthcare Centre, College of Clinical Medicine for Obstetrics & Gynecology and Pediatrics, Fujian Maternity and Child Health Hospital, Fujian Medical University, Fuzhou, 350001 China; 2 Department of Preventive Medicine, School of Public Health, Fujian Medical University, Minhou County, Fuzhou, 350122 China; 3 Department of Developmental and Behavioral Pediatrics, Fujian Children’s Hospital, Fujian, 350014 China

**Keywords:** Pediatric obesity, Hsa_circ_0000284, Hsa_circ_0046367, Neural plasticity mechanisms

## Abstract

**Objective:**

Our present study utilized case–control research to explore the relationship between specific circRNAs and pediatric obesity through a literature review and bioinformatics and to predict their possible biological functions, providing ideas for epigenetic mechanism studies of pediatric obesity.

**Methods:**

CircRNAs related to pediatric obesity were preliminarily screened by a literature review and qRT–PCR. CircRNA expression in children with obesity (n = 75) and control individuals (n = 75) was confirmed with qRT–PCR in a case–control study. This was followed by bioinformatics analyses, such as GO analysis, KEGG pathway analysis, and ceRNA network construction. Multivariate logistic regression was utilized to analyze the effects of circRNAs on obesity. A receiver operating characteristic (ROC) curve was also drawn to explore the clinical application value of circRNAs in pediatric obesity.

**Results:**

Has_circ_0046367 and hsa_circ_0000284 were separately validated to be statistically downregulated and upregulated, respectively, in the peripheral blood mononuclear cells of children with obesity and revealed as independent indicators of increased CHD risk [hsa_circ_0046367 (OR = 0.681, 95% CI: 0.480 ~ 0.967) and hsa_circ_0000284 (OR = 1.218, 95% CI: 1.041 ~ 1.424)]. The area under the ROC curve in the combined analysis of hsa_circ_0046367 and hsa_circ_0000284 was 0.706 (95% CI: 0.623 ~ 0.789). Enrichment analyses revealed that these circRNAs were actively involved in neural plasticity mechanisms, cell secretion and signal regulation.

**Conclusion:**

The present research revealed that low expression of hsa_circ_0046367 and high expression of hsa_circ_0000284 are risk factors for pediatric obesity and that neural plasticity mechanisms are closely related to obesity.

**Supplementary Information:**

The online version contains supplementary material available at 10.1186/s12887-023-04261-1.

## Introduction

The rate of overweight and obesity in children continues to increase rapidly, and this has become a major public health problem that affects the physical and mental health of children [1]. If children and adolescents suffer from overweight and obesity, the risk of suffering chronic diseases, unhealthy mental health and premature death in adulthood (such as type 2 diabetes and cardiovascular diseases) is increased [2]. The persistent increase in obesity rates worldwide will be one of the most difficult challenges facing humanity. Its pathogenesis is related to changes in adipose tissue metabolism, which is an important driver of many metabolic disorders and serious diseases, including type 2 diabetes (T2DM), cardiovascular disease (CVD) and certain types of cancer [3] [4]. Researchers have identified that obesity is a combined result of many factors, such as genetic, environmental and individual behavioral factors [3] [4]. However, the genetic pathology of obesity remains unclear.

Numerous studies have demonstrated that epigenetics (e.g., DNA methylation, microRNA [miRNA], long-chain non-coding RNA [lncRNA] and circular RNA [circRNA]) may play key roles in the progression of pediatric obesity [5–8]. CircRNAs are a class of evolutionally conserved and stable non-coding RNAs (ncRNAs) that are widely distributed in the nucleus and cytoplasm [9]. These characteristics make circRNAs potential biomarkers for various human diseases. Multiple studies have revealed that circRNAs can be involved in the inflammatory process [10], lipid metabolism [11] and endocrine changes [12]. Research has found that hsa_circH19 improves insulin sensitivity [13], and its abundance increases significantly with increases in obesity-related parameters [14], including body mass index (BMI), waist circumference, body fat percentage, and visceral fat area. However, the definite role of circRNAs in the progression of obesity is not very clear, and most previous studies have focused on the relationship between circRNAs and adult obesity.

To solve the limitations of the current study, we carried out a case–control study in southeastern China. In this study, we analyzed the differential expression of circRNAs in children with obesity and healthy control individuals by peripheral blood PCR experiments and further explored the relationship between circRNAs and obesity through software prediction and correlation analysis, which provides research clues for the epigenetic mechanism of pediatric obesity.

## Methods

### Patient and sample selection

**Fig. 1:**
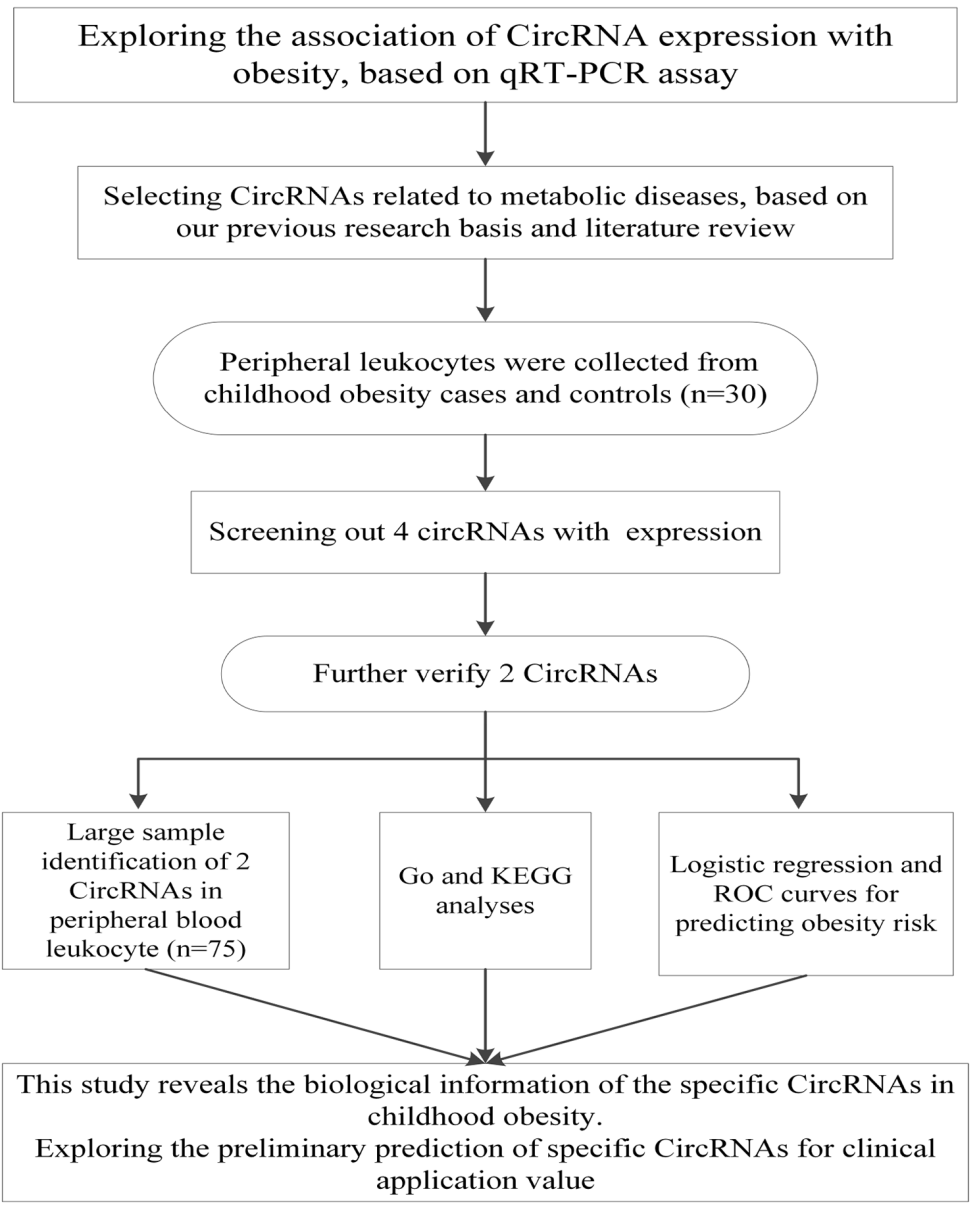
Illustrative figure of the study

CircRNAs related to metabolic diseases (glucose metabolism and lipid metabolism) were selected based on our previous research basis and literature review and are shown in Table S1. Figure 1 shows the illustrative feature of the study. qRT–PCR was performed in pediatric obesity cases and controls (n = 30) to screen out circRNAs with differential expression. Furthermore, differentially expressed circRNAs were selected for qRT–PCR verification in peripheral blood leukocytes (n = 75).

Data were collected from Fujian Maternity and Child Health Hospital, Affiliated Hospital of Fujian Medical University, between October 2019 and December 2020. Patients were eligible when (1) the “height/weight value” was ≥ 120% of the standard body weight, (2) the child/baby was 3–6 years old, (3) the patient did not suffer from congenital genetic diseases (e.g., congenital heart disease) or secondary obesity caused by metabolic diseases or neurological and endocrine diseases, (4) the health examination data was complete, and (5) the parents and children volunteered for the study with informed consent. All the research subjects signed informed consent forms. Our research protocol meets the Helsinki declaration, and the plan was approved by the Fujian Maternity and Child Health Hospital, Affiliated Hospital of Fujian Medical University Ethics Committee.

### Data collection

Data were collected from all the participants using face-to-face interviews with a structured questionnaire. Comprehensive data on sex, the one-child, support, total monthly household income, and relevant biochemical indicators from the health care system, including γ-glutamyl transpeptidase, alanine aminotransferase, triglycerides, total cholesterol, LDL, HDL, uric acid, glucose, calcium, potassium, phosphorus, chlorine, erythrocyte count, hematocrit, hemoglobin, platelet count, percentage of eosinophils, eosinophil count, percentage of neutrophils, and neutrophil count, were collected.

“Height/weight” is an indicator actively recommended by the WHO that focuses on reflecting the current nutritional status of children. In this study, the reference standard recommended by the WHO [15] was used to evaluate the physical development level of children by using this index. “Weight/ height” was obtained by physical measurement, and children with ≥ 110% of the standard weight were screened out. If the diagnosis by the child’s doctor at the health check-up is reassessed, the final diagnosis is made as follows: ≥110% and < 120% are overweight, and ≥ 120% are obese, excluding pathological or secondary obesity [16].

### qRT–PCR assay

The procedure for the separation of leukocytes using Human Peripheral Blood Lymphocyte Isolate (product number: LTS1077, www.tbdscience.com) is as follows. After 2 ml of lymphocyte isolate was placed in a 15 ml centrifuge tube, 2 ml of blood cells was slowly added along the tube with a pipette so that the blood could be seen in the upper layer and the isolate in the lower layer. After 20 min of centrifugation (room temperature, 400 g), the contents of the tube were divided into 4 layers. The upper layer is plasma, the second layer is the leucocyte layer, the third layer is the lymphocyte isolate layer, and the lower layer is the red blood cell layer. The second turbid layer of leucocytes was pipetted into a 1.5 ml EP tube, washed with 8 ml of phosphate buffer and centrifuged at 10,000 × g for 5 min at 4 °C. The supernatant was aspirated, and the leucocytes were retained. Then, 1 ml of TRIzol reagent was added to the cell pellet. This was mixed well until the cell clumps were completely dispersed and the solution was clear. The leukocytes were stored at -80 °C.

After the isolation of peripheral blood leukocytes from 75 case and control blood samples using Human Peripheral Blood Lymphocyte Isolates, we extracted total RNA with TRIzol reagent (Sigma, USA) according to the instructions of the kit, and the quality and integrity of the RNA samples were measured. Samples with an OD260/OD280 ratio between 1.8 and 2.0 were accepted. One microgram of RNA was reverse transcribed using the PrimeScript RT Reagent Kit with gDNA Eraser (RR037A, Takara Bio Inc., Shiga, Japan). Specific circRNAs were looked up in the circular RNA database circBase (http://www.circbase.org). Sequences were further designed (Shanghai Bio Inc.) and synthesized (Takara) based on the sequences, as detailed in Table S2. qRT–PCR was performed on a Light Cycler 480 real-time PCR system (Roche, Switzerland) using the SYBR® Premix Ex TaqTM II kit (RR820A, Takara Bio Inc., Shiga, Japan) for 40 cycles.

According to the SYBR® Premix Ex TaqTM II kit (RR820A, Takara Bio Inc., Shiga, Japan), a real-time PCR system (Lightcycler 480) was used for qRT–PCR. A 20 µl reaction system was prepared on ice according to Table S3. The real-time PCR amplification program settings are shown in Table S4. After the reaction, the melting curve and amplification curve of the real-time PCR were confirmed, and the next step of data processing and analysis detection was performed. Real-time fluorescence quantitative PCR data were used to represent the expression of endogenous genes by the 2^−ΔΔCt^ method.

### Construction of the circRNA-miRNA–mRNA ceRNA network

Based on the two large databases of circBank and the circRNA interactome, we conducted bioinformatics predictions on the downstream miRNAs and their sites that the two circRNAs may bind to after verification using various data analysis methods, such as miRanda and RNAhybrid. Moreover, miRNA indicators that may be related to pediatric obesity were screened out through an examination of the literature. Using the miRWalk2.0, TargetScan, miRDB, and miRecords databases, the target mRNAs downstream of miRNAs bound to specific circRNAs were predicted by examining relevant literature. Then, based on databases such as DIANA, miRanda, PicTar and PITA, we further predicted the downstream target genes of miRNA and constructed a circRNA-miRNA–mRNA ceRNA network.

### GO and KEGG pathway analysis

We performed GO analysis on the target genes predicted by bioinformatics and constructed meaningful annotations of genes and gene products. KEGG analysis of the target genes of the differentially expressed circRNAs was carried out to explore the signaling pathways and biochemical metabolic pathways that may play a role in the occurrence of pediatric obesity.

### Statistical analysis

The results for continuous variables with non-normal distributions were described by P_50_ (P_25_-P_75_) and compared using the Mann–Whitney U test. Spearman’s method was used to analyze the correlation between the relative expression of target circRNA and biochemical indicators. The results for discrete variables are shown as percentages (%), and differences in distribution are tested by chi-square (χ2). A multivariate unconditional logistic regression model was utilized to analyze the odds ratio (OR) and 95% confidence interval (95% CI) of circRNA to predict pediatric obesity. ROC curve analysis was conducted to determine the value of circRNAs.

## Results

### Screening for specific circRNAs

**Fig. 2:**
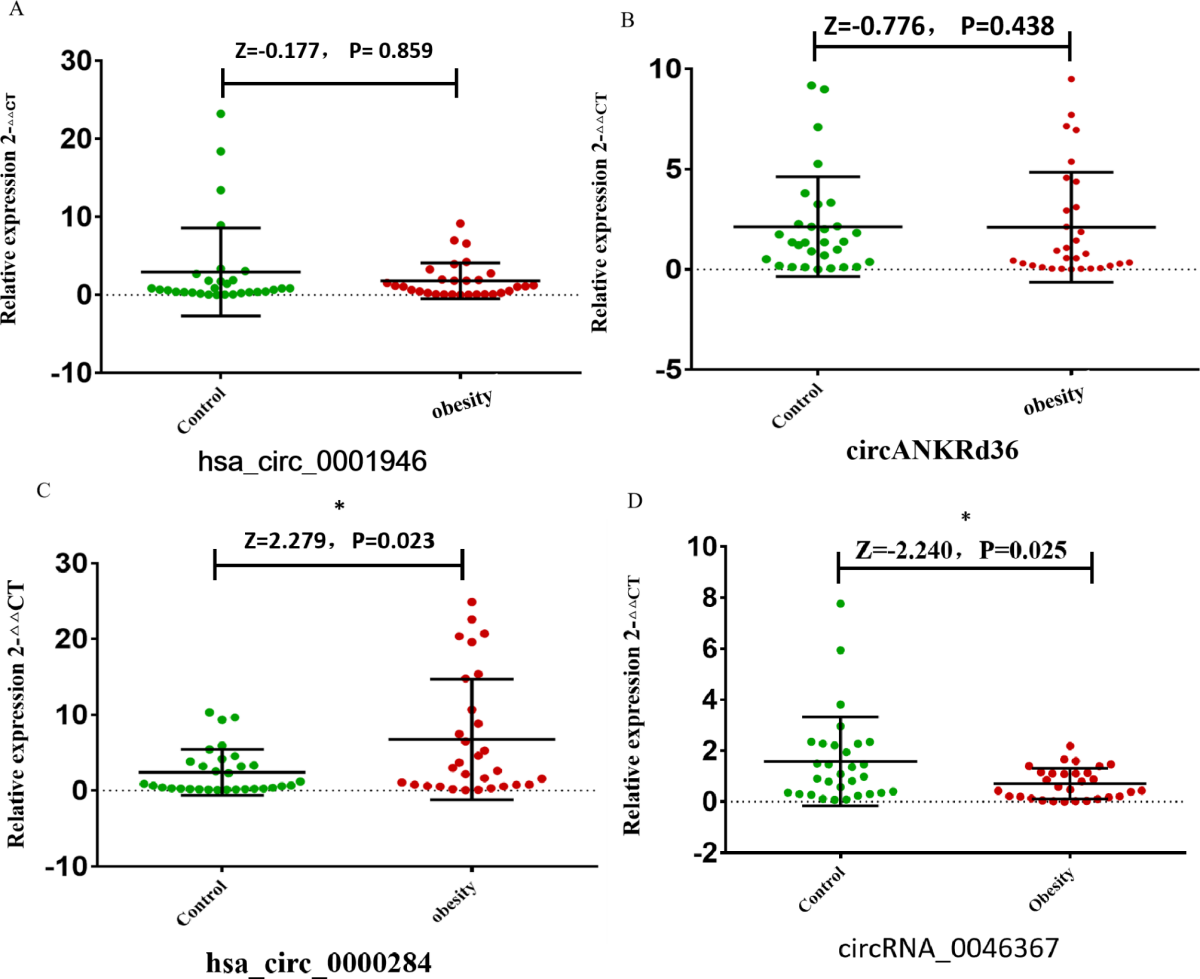
CircRNA expression levels in 30 cases and controls. A: hsa_circ_0001946; B: circANKRd36; C: hsa_circ_000284; D: circRNA_0046367. The 2^−ΔΔCt^ method was used to describe the expression of lncRNA.

Four differentially expressed circRNAs (hsa_circ_0001946, circANKRd36, hsa_circ_0046367 and hsa_circ_0000284) were identified through preliminary experiments. Candidate circRNAs were screened by qRT‒PCR in the peripheral blood leukocytes of 30 children with obesity and children of normal weight matched for age and sex. Their demographic characteristics are shown in Table S5. Figure 2 shows that the expression levels of hsa_circ_0000284 and hsa_circ_0046367 were significantly different between the two groups (P < 0.05). Finally, we selected these two circRNAs (hsa_circ_0000284 and hsa_circ_0046367) with different expression levels for follow-up research.

### Large sample verification of candidate circRNAs in peripheral blood leukocytes

**Fig. 3:**
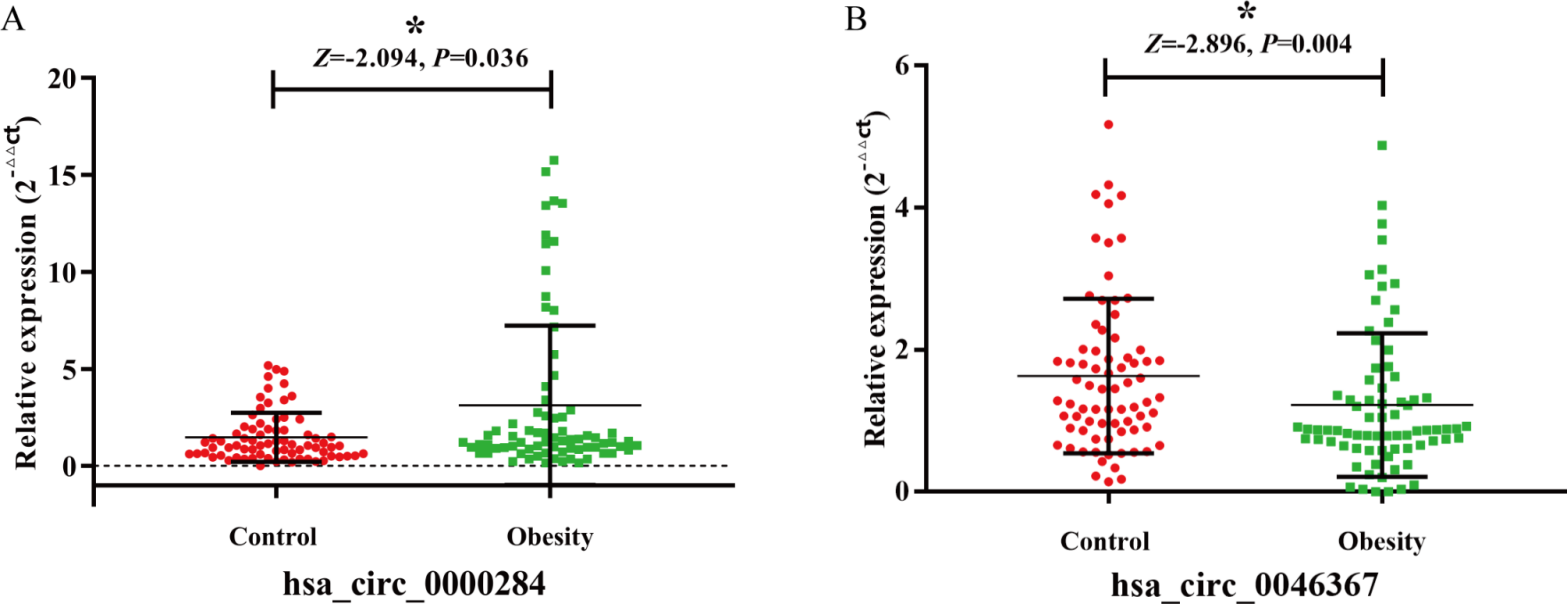
CircRNA expression levels in 75 cases and controls. A: hsa_circ_000284; B: circRNA_0046367. The 2^−ΔΔCt^ method was used to describe the expression of circRNA.

Next, 75 children with obesity and 75 children of normal weight were enrolled for an analysis of circRNA qRT‒PCR for further verification. Table 1 lists the characteristics of 75 children with obesity and children of normal weight. No significant difference was found in general demographic characteristics, such as sex and age (*P* > 0.05). Table S6 shows the differences in the levels of biochemical indicators between the pediatric obesity group and the control group. There were significant differences in the expression of hsa_circ_0000284 and hsa_circ_0046367 between the two groups. The expression level of hsa_circ_0000284 in the peripheral blood of children with obesity was higher than that in the control group, and the expression level of hsa_circ_0046367 in the peripheral blood of children with obesity was lower than that in the children in the control group (Fig. 3).

**Table 1:** Baseline characteristics of participants (n = 75)

Variables	Non-obesity	Obesity	*χ*^*2*^/t	*P* value
Sex			0.028	0.867
Male	46(61.3)	45(60.0)		
Female	29(38.7)	30(40.0)		
Age	5.013 ± 1.257	5.227 ± 1.169	0.1.076	0.284
The one-child			0.027	0.870
Yes	33(44.0)	34(45.3)		
No	42(56.0)	41(54.7)		
The supporter			0.132	0.716
parents	53(70.7)	55(73.3)		
grandparents	22(29.3)	20(26.7)		
Total monthly household income			0.401	0.818
low	11(14.7)	11(14.7)		
middle	51(68.0)	48(64.0)		
high	13(17.3)	16(21.3)		

### Analysis of the biological function of hsa_circ_0000284 and hsa_circ_0046367

**Fig. 4:**
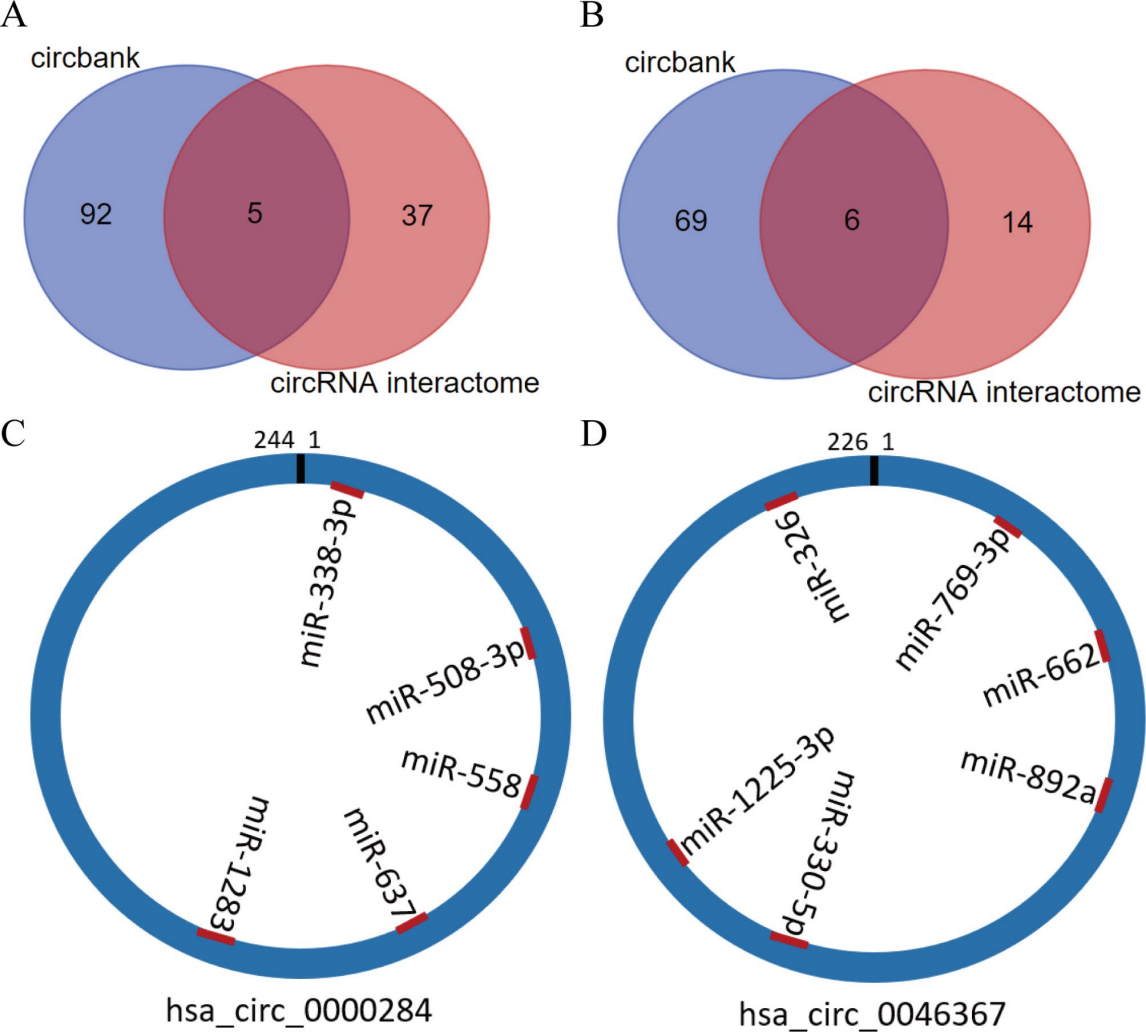
Target miRNAs bound by circRNAs predicted based on the circBank and circRNA interactome databases. A and B show the number of circRNA binding target miRNAs; C and D show the binding sites of circRNAs and target miRNAs.

Using the two major databases (e.g., circBank and circRNA interactome), we used analysis methods such as miRanda and RNAhybird to predict the bioinformatic function of these specific circRNAs. As shown in Table 2; Fig. 4, in the intersection of target miRNAs predicted by the above databases, hsa_circ_0000284 binds 5 miRNAs, and hsa_circ_0046367 binds 6 miRNAs. In addition, we predicted the binding sites of circRNA and target miRNAs (Fig. 4C-D).

**Table 2:** Target miRNAs bound by circRNAs

circRNA	miRNA
hsa_circ_0000284	hsa-miR-558
	hsa-miR-637
	hsa-miR-508-3p
	hsa-miR-1283
	hsa-miR-338-3p
hsa_circ_0046367	hsa-miR-330-5p
	hsa-miR-326
	hsa-miR-662
	hsa-miR-769-3p
	hsa-miR-892a
	hsa-miR-1225-3p

**Fig. 5:**
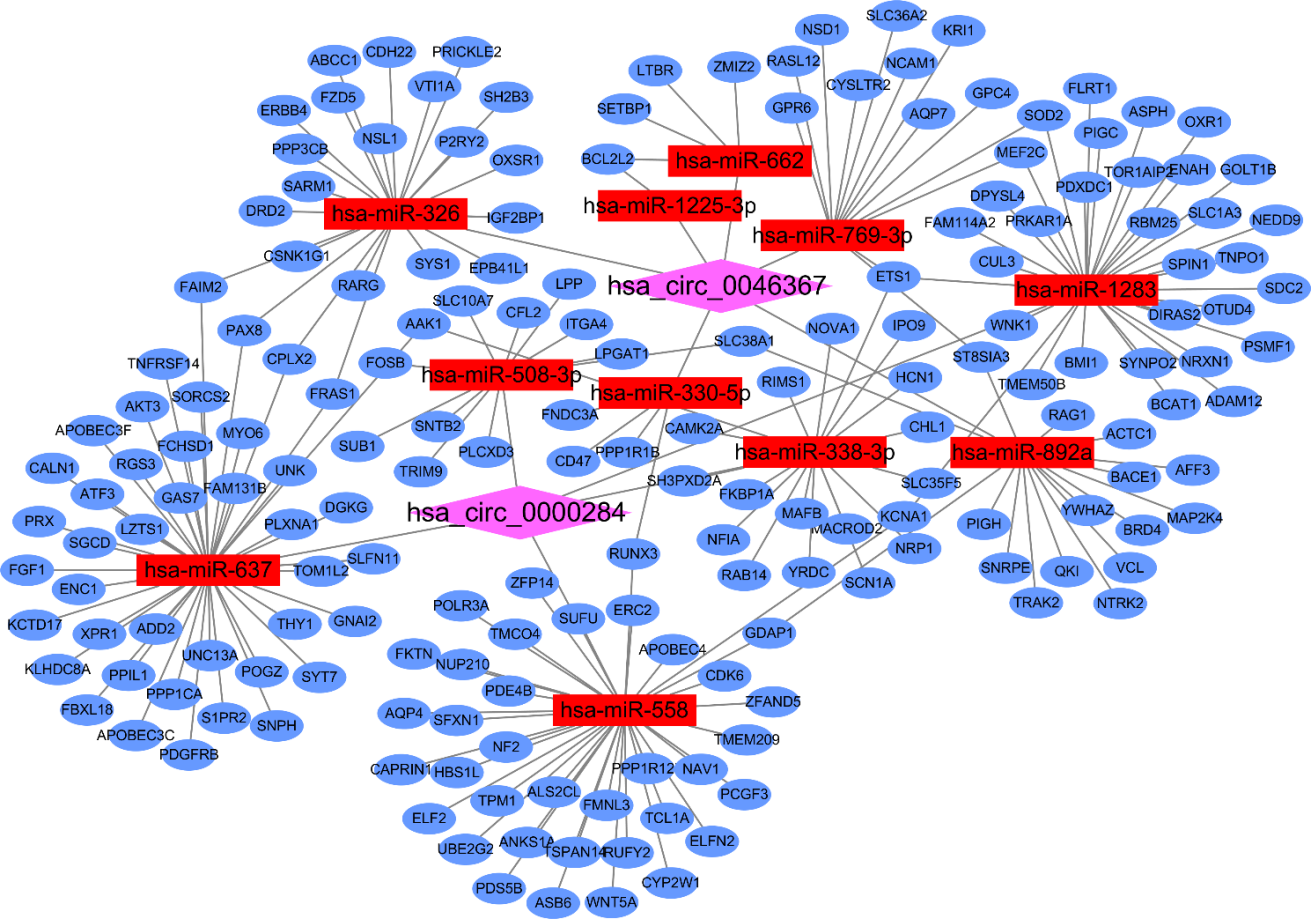
A circRNA-miRNA–mRNA ceRNA network

According to the miRNAs that hsa_circ_0000284 and hsa_circ_0046367 may bind to, we predicted downstream targeted mRNAs with regulatory relationships. Based on the literature and the results of bioinformatics analysis, an endogenous competitive RNA (ceRNA) regulatory network was constructed (Fig. 5). The network contains 2 circRNAs, 11 miRNAs and 185 mRNAs, suggesting that a significant correlation exists between the expression profiles of circRNAs and mRNAs.

**Fig. 6:**
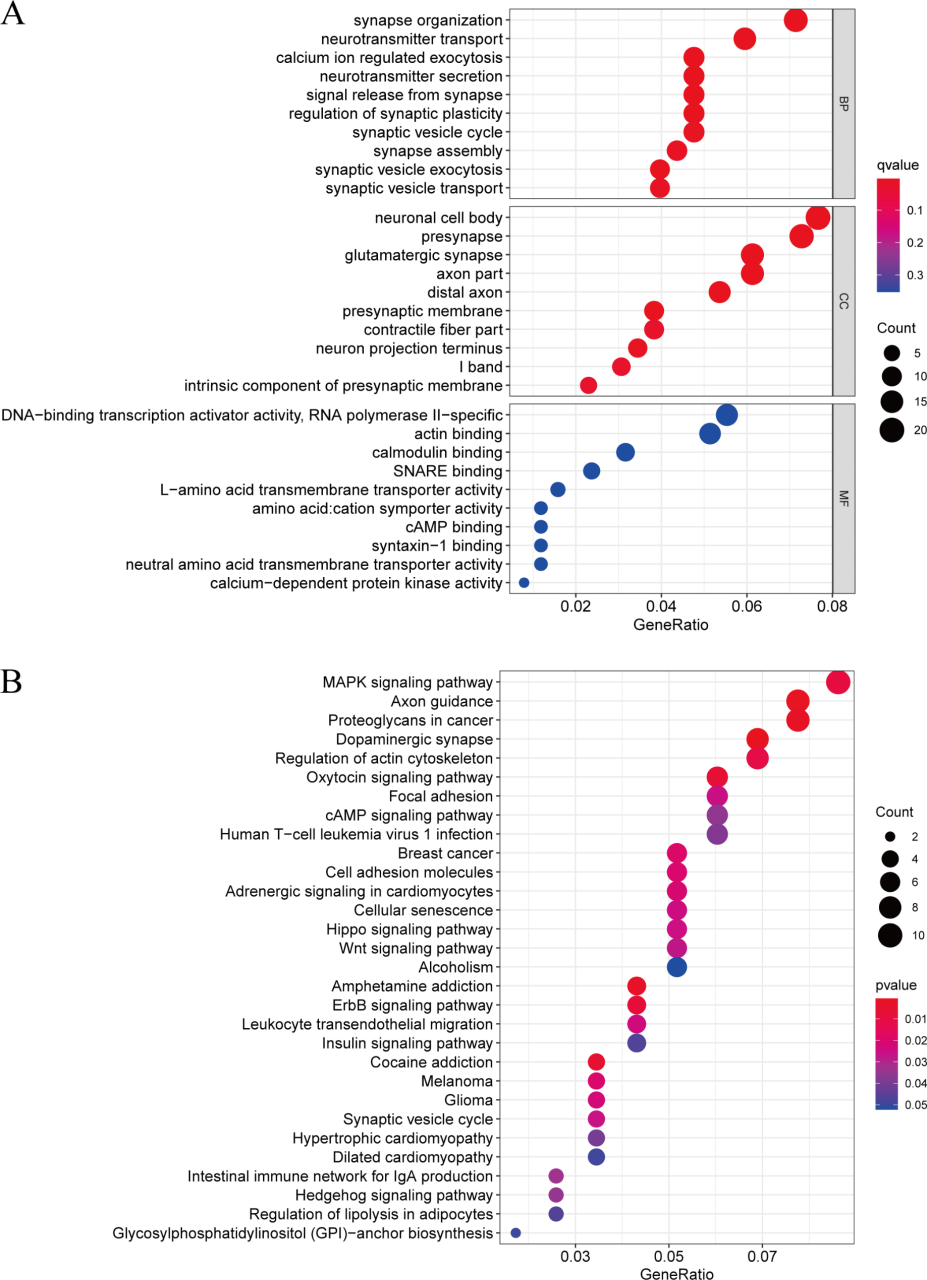
GO and KEGG enrichment analysis of circRNAs related to pediatric obesity

As shown in Fig. 6, GO analysis showed that the biological functions of hsa_circ_0000284 and hsa_circ_0046367 mainly included the “regulation of synaptic plasticity”, “neurotransmitter transportation”, “calcium ion regulation exocytosis”, and “signal release from synapse”. KEGG analysis [17–20] indicated that the MAPK signaling pathway was the most enriched functional term.

### Association between hsa_circ_0000284 and hsa_circ_0046367 and pediatric obesity

We further performed a multivariate logistic regression analysis to explore the effects of hsa_circ_0000284 and hsa_circ_0046367 on pediatric obesity (Table 3). The results revealed that high expression of hsa_circ_0000284 (OR = 1.267, 95% CI: 1.074 ~ 1.495) and low expression of hsa_circ_0046367 (OR = 0.685, 95% CI: 0.488 ~ 0.960) are risk factors for pediatric obesity.

**Table 3:** Logistic regression analyses of circRNAs and pediatric obesity

circRNA	*OR*	95% CI	*OR* ^a^	95% *CI*^a^
hsa_circ_0046367	0.682	0.491 ~ 0.946	0.685	0.488 ~ 0.960
hsa_circ_0000284	1.256	1.071 ~ 1.474	1.267	1.074 ~ 1.495

^a^Logistic regression (Enter). Variables entered into the model: sex, age, the one-child, support, and total monthly household income

**Fig. 7:**
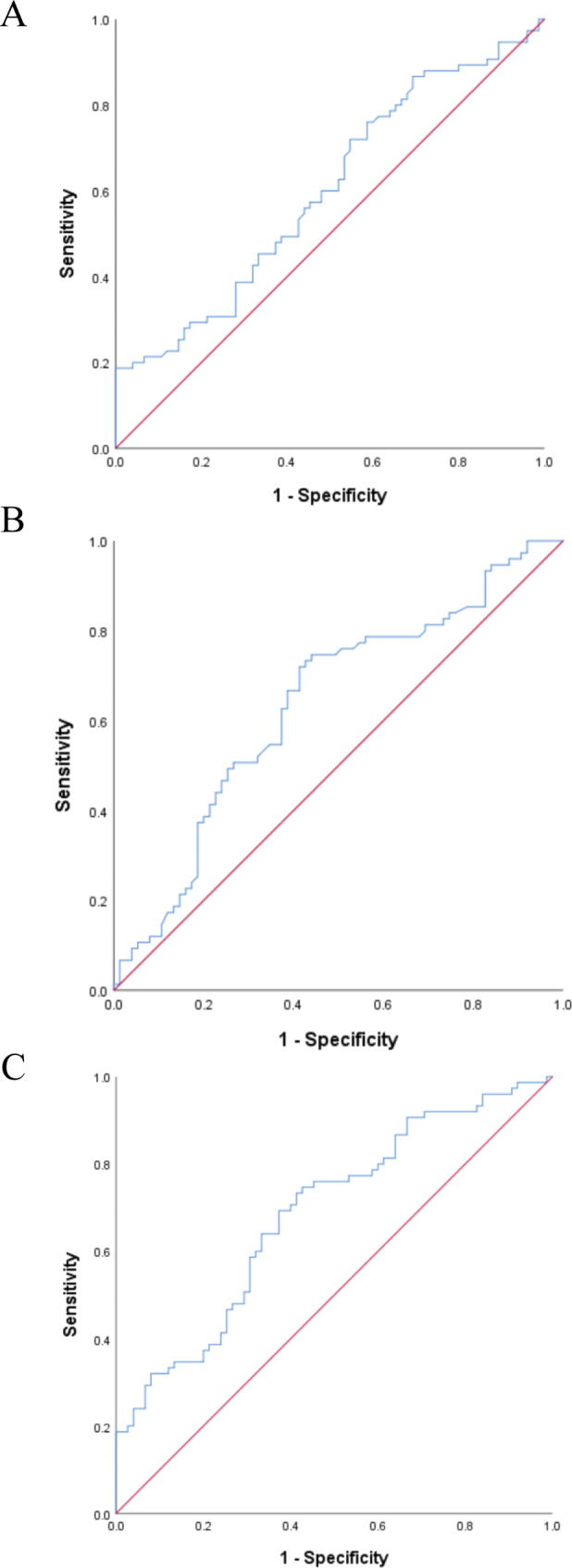
The ROC curve of circRNAs. A hsa_circ_000284; B circRNA_0046367; C the combination of hsa_circ_000284 and circRNA_0046367.

An ROC curve was carried out to evaluate the diagnostic sensitivity and specificity of hsa_circ_0000284 and hsa_circ_0046367 for pediatric obesity (Table S7 and Fig. 7). The area under the curve of hsa_circ_0046367 was 0.637 (95% CI: 0.547~0.727). The AUC of hsa_circ_0000284 was 0.599 (95% CI: 0.508~0.690). The AUC value reached 0.706 (95% CI: 0.623~0.789) when hsa_circ_0046367 and hsa_circ_0000284 were added simultaneously. This value was higher than that of a single indicator, but it was not statistically significant.

### The correlation between circRNAs and biochemical indicators

Moreover, we also focused on the correlation between circRNAs and biochemical indicators, as shown in Table 4. Spearman rank correlation analysis revealed that the expression level of hsa_circ_0046367 correlated with γ-glutamyl transpeptidase and that hsa_circ_0000284 correlated with eosinophil count and percentage of neutrophils.

**Table 4:** Spearman correlation analysis of circRNAs and biochemical indicators

Biochemical indicators	hsa_circ_0046367			hsa_circ_0000284		
	rs	P value		rs	P value	
γ-Glutamyl transpeptidase	**-0.166**	**0.042**		0.055	0.503	
Alanine aminotransferase	-0.159	0.051		0.114	0.164	
Triglycerides	0.035	0.671		0.106	0.197	
Total cholesterol	-0.015	0.860		0.015	0.856	
LDL	-0.075	0.359		0.025	0.758	
HDL	0.109	0.183		-0.098	0.235	
Uric acid	-0.038	0.644		0.096	0.243	
Glucose	0.002	0.985		-0.028	0.732	
Calcium	-0.073	0.374		-0.058	0.480	
Potassium	-0.117	0.152		-0.011	0.891	
Phosphorus	-0.133	0.104		0.055	0.500	
Chlorine	-0.055	0.505		0.004	0.961	
Erythrocyte count	-0.042	0.609		-0.157	0.056	
Hematocrit	-0.099	0.229		-0.038	0.644	
Hemoglobin	-0.091	0.270		0.0.046	0.575	
Platelet count	-0.113	0.169		0.052	0.528	
Percentage of eosinophils	0.040	0.627		-0.137	0.095	
Eosinophil count	-0.014	0.865		**-0.203**	**0.013**	
Percentage of neutrophils	0.003	0.972		**0.188**	**0.021**	
Neutrophil count	-0.007	0.930		0.082	0.321	

## Discussion

Several studies have disclosed that circRNAs play a regulatory role in the biological processes of obesity-related adipogenesis and differentiation and obesity-induced inflammation. Multivariate regression analysis revealed that low expression of hsa_circ_0046367 and high expression of hsa_circ_0000284 are risk factors for pediatric obesity. Researchers found that hsa_circ_0000284 is a product of splicing of exon 2 of the HIPK3 gene with 1099 nucleotides. It plays a role in regulating cell growth and can bind multiple miRNAs [20]. In addition, hsa_circ_0000284 is also involved in the biological processes of a variety of diseases, such as diabetes [21], diabetic retinopathy [22], age-related cataracts [23] and various types of cancer [24, 25]. Some studies indicate that circRNA_0046367 plays a regulatory role in lipid metabolism [26]. In patients with non-alcoholic fatty liver disease, circRNA_0046367 can regulate liver steatosis and oxidative stress through the circRNA_0046367/miR-34a/PPARα pathway, thereby improving hepatotoxicity caused by lipid peroxidation [26].

To explore the clinical application value of hsa_circ_0046367 and hsa_circ_0000284 in pediatric obesity, ROC analysis was used, and it suggested that hsa_circ_0046367 and hsa_circ_0000284 have certain clinical application values in the study of pediatric obesity. Due to the complexity of the pathogenesis of obesity, relying on a single circRNA model to predict and treat obesity may not provide great clinical practical value. Instead, a multi-marker approach combining multiple candidate circRNAs, miRNAs, and RNA-binding proteins (RBPs) should be considered; this approach may be more discriminative, accurate, and effective in predicting obesity and metabolic disorders [27].

Notably, hsa_circ_0046367 and hsa_circ_0000284 were applied as biological indicators that may affect pediatric obesity in the abovementioned studies, and the mechanism of their participation in the occurrence and development of pediatric obesity has not yet been reported. We found that circRNAs may be involved in neural plasticity mechanisms (e.g., “regulation of synaptic plasticity”, “neurotransmitter transportation”) and cell secretion and signal regulation mechanisms, as determined by bioinformatics technology. This indicated that circRNAs are crucial to the pathogenesis of pediatric obesity. The possible explanations for these results might be related to the findings of several studies that demonstrated that circRNAs are important regulators involved in neural plasticity mechanisms [28, 29], cell secretion [30] and cell signal regulation [31, 32]. Moreover, current studies have shown that the formation of obesity may share similar neuroplastic mechanisms with addictive drug abuse; that is, reward disorders induce disturbances in related synaptic plasticity [33]. Reward disorders induce the destruction of AMPARs to cause obesity; abnormalities in AMPAR expression, AMPAR transport and calcium conductance at glutamatergic synapses can trigger excitatory synaptic plasticity [34, 35].

We constructed a coexpression network based on two candidates. There are many circRNA-miRNA pairs in the coexpression network that may be involved in the mechanism of pediatric obesity. Among the 6 miRNAs bound by hsa_circ_0046367, hsa-miR-769-3p can bind PNPLA3 (adiponectin 3) to promote liver lipid synthesis [36]. Among the 5 miRNAs bound by hsa_circ_0000284, hsa-miR-637, hsa-miR-338-3p and miR-192-5p could be involved in immunity/inflammation [37], insulin resistance [12], and fatty acid metabolism [38], which are crucial to the pathogenesis of obesity. GO analysis results showed that target circRNAs were correlated with calmodulin binding. Calprotin can stimulate Toll-like receptor 4 (TLR4) and downstream inflammatory signaling pathways, promote the release of interleukin-6 (IL-6) and tumor necrosis factor-α (TNF-α), and act as an inflammatory signal amplifier [39]. It has been found that patients with coronary artery disease (CAD) had significantly increased serum IL-6 and TNF-α levels compared to control individuals [40].

Bioinformatics analysis of the corresponding gene pairs revealed that circRNAs are associated with the “mitogen-activated protein kinase (MAPK) signaling pathway”, “axon guidance”, “proteoglycans in cancer”, “dopaminergic synapses”, and “regulation of actin cytoskeleton”. Among them, the MAPK signaling pathway was the most enriched functional term. MAPK is an important signaling pathway in cells that is widely present in mammals, and it can phosphorylate a variety of protein kinases and nuclear transcription factors. It can be activated by different stimuli or signals, such as growth factors and physical stress, to produce different transduction pathways and target and activate different transcription factors to regulate cell proliferation, differentiation, apoptosis, intercellular functional conduction and other biological and physiological processes [41, 42]. In addition, MAPK can play an important regulatory role in the process of adipocyte differentiation [43, 44]. In the predicted MAPK pathway, the target circRNA was closely related to ELK1. ELK1 is an ETS domain transcription factor associated with adipocyte differentiation. As a miRNA target, phosphorylation of ELK1 activates many other transcription factors and plays an important regulatory role in the differentiation of visceral fat [45, 46]. The MAPK signaling pathway may be an important mechanism in the occurrence of obesity, which will be further studied in the future.

In addition, we also found that the expression level of hsa_circ_0046367 is correlated with γ-glutamyl transpeptidase and that hsa_circ_0000284 is correlated with inflammation indicators (eosinophil count, neutrophil percentage). There is a negative correlation between hsa_circ_0046367 and γ-glutamyl transpeptidase. The concentration of γ-glutamyl transpeptidase can reflect the degree of liver damage to a certain extent. The decrease or loss of hsa_circ_0046367 expression will aggravate liver cell steatosis and lipid peroxidation, leading to fatty liver [26]. This suggests that hsa_circ_0046367 is related to the lipid metabolism of pediatric obesity. This finding also suggests some new ideas for the mechanisms underlying the relationships between specific circRNAs and pediatric obesity.

There are limited data on obesity and circRNA in preschool-age children in terms of the number of studies available, but our study adds to the body of relevant research evidence in this area. We also added a raw letter analysis of the corresponding circRNAs to predict their possible biological functions and to provide ideas for the study of epigenetic mechanisms of childhood obesity. However, some limitations should be noted. First, causality is difficult to demonstrate in a case‒control design, although there are multiple theories supporting our findings. Second, the sample size of our study is small because of the technical demands of drawing blood from children and the difficulty of drawing blood from children in China, where everyone in the family takes great care of them. In addition, because there are relatively few studies on the association between circRNAs and obesity in preschool children, we selected circRNAs associated with metabolic diseases (glucose metabolism and lipid metabolism) from the literature for further experimental studies. Subsequent transcriptome sequencing and construction of ROC curve analysis of the circRNAs-miRNA‒target gene axis should be considered for good clinical utility.

## Conclusion

In conclusion, our research facilitates the understanding that low expression of hsa_circ_0046367 and high expression of hsa_circ_0000284 are independent risk factors for pediatric obesity. These findings reveal that circRNAs might be involved in the biological process of pediatric obesity and indicate the close relationship between neural plasticity mechanisms and obesity to provide new ideas for research on the mechanisms of pediatric obesity.

### Electronic supplementary material

Below is the link to the electronic supplementary material.

Supplementary Material 1

## Data Availability

Participant-level data are available from the corresponding author at lgb0703@163.com.

## Abbreviations

CircRNAs: Circular RNAs
miRNAs: microRNAs
ncRNAs: Non-coding RNAs
BMI: Body mass index
OR: Odds ratio
CI: Confidence interval
ROC: Receiver operating characteristic
AUC: Area under the curve
