# Supplementary material for: Exploring the association between circRNA expression and pediatric obesity based on a case–control study and related bioinformatics analysis
Source: BMC Pediatr. 2023 Nov 13;23:561. doi: 10.1186/s12887-023-04261-1 (PMC10642011; doi:10.1186/s12887-023-04261-1)
Supplement: Supplementary file 1 — Supplementary Material 1 [file 12887_2023_4261_MOESM1_ESM.pdf]

Table S1 CircRNAs related to obesity, glucose metabolism and lipid metabolism

CircRNA	Potential biological functions and phenotypes	Reference
CircWDR77	The CircRNAWDR77-miR-124-FGF2 axis regulates the proliferation and migration of vascular smooth muscle cells (VSMCs) under high-glucose conditions.	[1]
hsa_circ_0046367	The circRNA_0046367/miR-34a/PPARα axis promoted steatosis in HepG2 cells induced by high lipid mixture.	[2] [3]
hsa_circ_0046366	Hepatic steatosis was inhibited by miR-34a/PPARα signaling.	[4] [5]
CircScd1	Steatosis in non-alcoholic fatty liver disease (NAFLD) is influenced by the JAK2/STAT5 signaling pathway.	[6]
Hsa_circ_0054633	It is associated with prediabetes and T2DM.	[7] [8]
circANKRd36	It associated with chronic inflammation in patients with T2DM.	[9]
hsa_circ_0000284 (circRNAHIPK3）	β-cell function was controlled by isolating miR-124.3p/miR-138.3p.	[10] [11] [12]
hsa_circ_0001946 （CirRS-7）	Targeted binding of miR-7 in mouse β cells increased insulin secretion.	[10] [13] [14]

[1] Westholm J O, Miura P, Olson S, et al. Genome-wide analysis of drosophila circular RNAs reveals their structural and sequence properties and age-dependent neural accumulation [J]. Cell Rep, 2014, 9(5): 1966-1980.

[2] Xing-Ya G, Jian-Neng C, Fang S, et al. circRNA_0046367 Prevents Hepatoxicity of Lipid Peroxidation: An Inhibitory Role against Hepatic Steatosis [J]. Oxidative Medicine & Cellular Longevity, 2017, 17(7): 3960197.

[3] Guo J, Zhou Y, Cheng Y, et al. Metformin-Induced Changes of the Coding Transcriptome and Non-Coding RNAs in the Livers of Non-Alcoho lic Fatty Liver Disease Mice [J]. Cell Physiol Biochem, 2018, 45(4): 1487-1505.

[4] Guo X Y, Sun F, Chen J N, et al. circRNA_0046366 inhibits hepatocellular steatosis by normalization of PPAR signaling [J]. World Journal of Gastroenterology, 2018, 24(3): 323-337.

[5] Armstrong M J, Gaunt P, Aithal G P, et al. Liraglutide safety and efficacy in patients with non-alcoholic steatohepatitis (LEAN): a multicentre, double-blind, randomised, placebo-controlled phase 2 study [J]. Lancet, 2016, 387(10019): 679-690.

[6] Li P, Shan K, Liu Y, et al. CircScd1 Promotes Fatty Liver Disease via the Janus Kinase 2/Signal Transducer and Activator of Trans cription 5 Pathway [J]. Dig Dis Sci, 2019, 64(1): 113-122.

[7] Zhao Z, Li X, Jian D, et al. Hsa_circ_0054633 in peripheral blood can be used as a diagnostic biomarker of pre-diabetes and type 2 diabetes mellitus [J]. Acta Diabetol, 2017, 54(3): 237-245.

[8] Yang F, Liu D Y, Guo J T, et al. Circular RNA circ-LDLRAD3 as a biomarker in diagnosis of pancreatic cancer [J]. World J Gastroenterol, 2017, 23(47): 8345-8354.

[9] Fang Y, Wang X, Li W, et al. Screening of circular RNAs and validation of circANKRD36 associated with inflammation in patients wit h type 2 diabetes mellitus [J]. Int J Mol Med, 2018, 42(4): 1865-1874.

[10] Stoll L, Sobel J, Rodriguez-Trejo A, et al. Circular RNAs as novel regulators of β-cell functions in normal and disease conditions [J]. Mol Metab, 2018, 9(3): 69-83.

[11] Bao X, He X, Zheng S, et al. Up-regulation of circular RNA hsa_circ_0037909 promotes essential hypertension [J]. J Clin Lab Anal, 2019, 33(4): e22853.

[12] Shan K, Liu C, Liu B H, et al. Circular Noncoding RNA HIPK3 Mediates Retinal Vascular Dysfunction in Diabetes Mellitus [J]. Circulation, 2017, 136(17): 1629-1642.

[13] Xu H, Guo S, Li W, et al. The circular RNA Cdr1as, via miR-7 and its targets, regulates insulin transcription and secretion in islet cells [J]. Sci Rep, 2015, 5(6): 12453.

[14] Lightell D J, Moss S C, Woods T C. Upregulation of miR-221 and -222 in response to increased extracellular signal-regulated kinases 1/2 activity exacerbates neointimal hyperplasia in diabetes mellitus [J]. Atherosclerosis, 2018, 269(3): 71-78.

Table S2 Primer sequences of circRNAs and reference genes

The name of the gene	Primer	The sequence（5'.3'）
GAPDH (endogenous gene)	Forward	GAAGGTGAAGGTCGGAGTCAAC
	Reverse	CAGAGTTAAAAGCAGCCCTGGT
circRNA_010567	Forward	CAGCGTCCTTTCTCAAGGGA
	Reverse	GACCTGATTGGCCACTCAGTA
circWDR77	Forward	TCCAGCAACAGGACGAAATG
	Reverse	TGGAGATCCTCGGACTGGAA
hsa_circ_0046367	Forward	CTCGCTTCGGCAGCACA
	Reverse	AACGCTTCACGAATTTGCGT
hsa_circ_0046366	Forward	CGTCCATTCGTTTGTGAGCC
	Reverse	CTTCACAGCCTCATCGGAGC
circScd1	Forward	CACGACCCCACCTATCAGGA
	Reverse	GAGATCGAGCGTGGACTTCG
hsa_circ_0054633	Forward	CCAATATTGTATAACTAGCTCCTC
	Reverse	GCACTTTATTAGATTACAGTATC
circANKRd36	Forward	GGAGGCCACAAGTGATGAGA
	Reverse	CCTGGTGGTTTCTCAGAAGAC
hsa_circ_0000284	Forward	GTGAACAAGTCCAAGAGGACC
	Reverse	ACAGATGCTCTTCAAGGATGC
hsa_circ_0001946	Forward	AGTCTTCCATCAACTGGCTCA
	Reverse	GACACAGGTGCCATCGGA

Table S3 CircRNA real-time fluorescence quantitative PCR reaction system

Reagent	volume
PCR Forward Primer (10 μM)	0.8μl
PCR Reverse Primer (10 μM)	0.8μl
TBGreen PremixExTaqII (2x)	10.0μl
RNase Freed H2O	6.4μl
cDNA	2.0μl
Total reaction volume	20.0μl

Table S4 Real-time PCR amplification procedure for circRNAs

Reaction stage	Temperature (℃)	Time	Number of cycles
Predegradation	95	30s	1
PCRreaction	95	1s	45
	60	34s	45
Melting reaction	95	15s	1
	60	1min	1
	95	15s	1

Table S5 Baseline characteristics of participants (n=30)

**Variables**	**Non-obesity**	**Obesity**	χ^2/t	***P* value**
Sex			3.360	0.067
Male	21(70.0)	14(46.7)		
Female	9(30.0)	16(53.3)		
Age	5.167±1.147	4.767±1.278	.1.028	0.207
The one-child			1.763	0.184
Yes	9(30.0)	14(46.7)		
No	21(70.0)	16(53.3)		
The supporter			0.077	0.781
parents	20(66.7)	21(70.0)		
grandparents	10(33.3)	9(30.0)		
Total monthly household income			1.709	0.426
low	5(16.7)	2(6.7)		

middle	21(48.8)	22(73.3)
high	4(13.3)	6(20.0)

Table S6 The level of biochemical indicators of participants (n=75)

Biochemical indicators	Non-obesity	Obesity	*Z* value	*P* value
γ-Glutamyl transpeptidase	12.80(11.35-15.00)	16.40(13.70-20.90)	-5.357	＜0.001
Alanine aminotransferase	13.50(11.45-17.05)	18.10(13.70-23.50)	-4.858	＜0.001
Triglycerides	0.89(0.67-1.23)	1.03(0.76-1.47)	-2.227	0.026
Total cholesterol	4.45(4.11-4.94)	4.66(4.14-5.22)	-1.154	0.249
LDL	2.51(2.13-2.93)	2.73(2.37-3.25)	-2.299	0.022
HDL	1.46(1.27-1.68)	1.33(1.19-1.51)	-2.502	0.012
Uric acid	291.30(255.15-338.05)	344.60(302.30-396.10)	-4.022	＜0.001
Glucose	4.93(4.59-5.23)	5.02(4.80-5.23)	-1.522	0.128
Calcium	2.48(2.43-2.53)	2.51(2.44-2.55)	-1.832	0.067
Potassium	4.30(4.20-4.50)	4.40(4.20-4.60)	-1.683	0.092
Phosphorus	1.54(1.49-1.68)	1.59(1.49-1.65)	-1.102	0.271
Chlorine	105.70(104.90-107.45)	106.10(104.80-107.80)	-0.622	0.534
Erythrocyte count	4.73(4.52-5.00)	4.74(4.56-4.95)	-0.571	0.568
Hematocrit	37.90(36.05-39.50)	38.60(37.40-40.50)	-2.499	0.012
Hemoglobin	129.00(124.50-135.00)	134.00(128.00-138.00)	-2.983	0.003
Platelet count	300.00(268.00-331.00)	319.00(274.00-352.00)	-1.866	0.062

Percentage of eosinophils	2.40(1.30-4.35)	2.60(1.60-3.90)	-0.294	0.769
Eosinophil count	0.19(0.10-0.29)	0.19(0.11-0.30)	-0.179	0.858
Percentage of neutrophils	42.00(34.25-51.05)	45.4(36.40-52.60)	-1.515	0.130
Neutrophil count	2.92(2.16-4.03)	3.09(2.51-4.28)	-1.413	0.158

Table S7 Sensitivity and specificity of circRNA for predicting childhood obesity

circRNA	AUC(95% CI)	Sensitivity	Specificity	Youden's index	Positive predictive value	Positive predictive value
hsa_circ_0046367	0.637 (0.547~0.727)	0.747	0.560	0.307	0.112	0.968
hsa_circ_0000284	0.599 (0.508~0.690)	0.760	0.413	0.187	0.088	0.959
Conjoint analysis	0.706 (0.623~0.789)	0.760	0.627	0.387	0.132	0.972
